# Macroeconomic dynamics under bounded rationality: on the impact of consumers’ forecast heuristics

**DOI:** 10.1007/s11403-022-00348-7

**Published:** 2022-03-05

**Authors:** Tae-Seok Jang, Stephen Sacht

**Affiliations:** 1 Department of Economics, Kyungpook National University, 80 Daehak-ro, Buk-gu, Daegu, 41566 Republic of Korea; 2 Department of Economics, Kiel University, Olshausenstrasse 40, 24118 Kiel, Germany

**Keywords:** Bounded rationality, Consumer confidence, Forecast heuristics, Impulse response analysis, New Keynesian model, C53, D83, E12, E21, E32

## Abstract

This paper considers various types of forecast heuristics to examine the effects of boundedly rational agents on macroeconomic dynamics. Given the baseline New Keynesian model, we seek to find the expectation formation process that is most suitable in describing economic adjustments over the business cycle. In particular, impulse response analysis is used to compare the performances of the macroeconomic model under bounded rationality and under rational expectations. The results show that the fluctuations in consumer confidence mainly explain the degree of persistence in consumption. We conclude that a model under bounded rationality with a heuristic-induced switching process can qualitatively provide a good fit to the data that is equivalent to a model under rational expectations.

## Introduction

Modern macroeconomic models often rely on the rational expectations (RE) hypothesis to avoid the complexity of multiperiod optimization problems in economic activities. Under so-called perfect rationality, the optimal paths for consumption and investment can be obtained (or at least approximated) if no further complications from unforeseen problems occur. The optimal solutions are specific to individuals and policymakers who try to navigate through business cycles, but they come at the expense of ignoring potential unexpected crises.

Thus, an alternative to perfect rationality has always been needed. As the field of behavioral and experimental economics gradually matures, the notion of bounded rationality (BR) provides a benchmark against which agents attempt to forecast events. For example, heuristics, such as rules-of-thumb procedures, are applied as a convenient way to describe the reality and complexity of economic activities. This is because the economic agents who observe the structure of the economy barely understand the interactions between relevant variables, such as output and inflation (Munier et al. 1999). Given the lack of full information, such bounded rational agents rely on habits, imitation, and/or procedural optimization to predict changes in the economy (Day and Pingle 1991).

Perhaps the most prominent type of heuristics found in the recent macroeconomic analysis literature is based on discrete choice models.^1^ In some cases, heuristic expectation formation might be governed by a switching process under the consideration of heterogeneous forecasting rules (Jang and Sacht (2016, 2021)). In particular, agents sort themselves into different groups, and each group is populated by individuals who have strong beliefs in a certain expectation formation process. As a result, endogenous waves of economic beliefs, such as optimism and pessimism, are generated from period to period. This leads to fluctuations in the economic variables driven by reversals in the emotional state, which holds even in the absence of autocorrelated exogenous shocks.

The theoretical underpinnings of heuristics have also received support from empirical and experimental studies. For example, the experimental results of the work by Cornand and Heinemann (2018) suggest that the simplest form of adaptive (i.e., extrapolative) heuristics provides a better description of reality. Grazzini and G. R. and Tsionas, M. (2017) explicitly consider the heuristics of fundamentalists and chartists applied to the output gap expectation when estimating a DSGE model via Bayesian techniques. Meanwhile, Assenza et al. (2013) conduct a laboratory experiment on a purely forward-looking new Keynesian model (NKM) where the discrete choice approach is applied. The results support the adaptive process in forecast heuristics and a slow convergence to the steady-state output level. A comprehensive review of expectation formations in macroeconomics can be found in Assenza et al. (2013) and Franke and Westerhoff (2018).

In this paper, we contribute to the empirical literature on behavioral economics by examining the effects of bounded rationality on macroeconomic dynamics. As a continuation of the work by Jang and Sacht (2021), we consider various types of forecast heuristics that connect consumer confidence and private household expenditure. In particular, the authors investigated the role of consumer confidence in the determination of household expenditure and its influence on the business cycle. As a key feature of the BR model framework, agents are allowed to switch to the group with the best performing forecast strategy as evaluated based on discrete choice theory. Their results suggest that expectations in the US economy are grounded on the consumers’ emotional state, whereas in the Euro Area, they are purely technical. In the latter case, the fundamentalists’ and chartists’ heuristics model under consideration exhibits the best possible description of the data. The study shows that consumer confidence plays a crucial role in the determination of household expenditure and the pass-through to GDP fluctuations.

The importance of confidence in decision-making processes motivates the research question regarding the effects of the occurrence of certain shocks to macroeconomic dynamics via confidence. In this paper, as an investigation of forecast heuristics, we go beyond Jang and Sacht (2021) and analyze the impulse response functions (IRFs) to various shocks to economic variables. To observe this, we select the specification in the heuristics that leads to the best description of different versions of a standard DSGE model from the empirical data. In doing so, we compare the IRFs stemming from the BR model with the ones obtained from the RE model. Both types of IRFs are confronted with the outcome of a vector autoregressive (VAR) model.

We note that such analysis is of high interest, especially in the case of a potential output/inflation trade-off that the central bank will face when conducting an (optimal) monetary policy intervention. It is also worthwhile to examine the way persistence is introduced into the model. This is crucial to capture the degree of inertia being observed for the empirical time series induced by the statistical VAR model. Both theoretical models handle the issue differently. While under RE, intrinsic persistence results from the assumption of habit formation in consumption and price indexation, backward-looking elements are directly incorporated into the heuristics being applied under BR. As a novel feature, we show that the macroeconomic dynamics driven by heuristics can provide a good fit to the data that is equivalent to a RE model with a lead and lag structure.

Indeed, our results address the challenges that policymakers face, especially when stimulating the economy via fiscal and/or monetary policy in the presence of ‘animal spirits’. For example, few studies have investigated the (optimal) monetary and fiscal policy under BR (cf. Caprioli 2015; De Grauwe and Macchiarelli 2015; Hollmayr and Matthes 2015). Cornea-Madeira et al. (2019) state that valid empirical evidence for behavioral heterogeneity is questioning the formulation of the optimal policy design under the RE paradigm because of the existence of multiple equilibria in a complex system under BR. They show that heterogeneity varies over time and conclude that inflation dynamics can be dominated by either forward-looking or backward-looking behavior. Also, Lengnick (2016) discuss the design and implementation of optimal simple rules in the baseline NKM with an additional financial sector included under the discrete choice switching mechanism. They show that considering a simple Taylor rule under BR (i.e., limited to specific types of fundamentalists and chartists) causes a strong increase in the central bank’s objective function. The result is explained by the fact that monetary policy rules must be more forward-looking as the model becomes more backward-looking (cf. Leitemo 2008).

The remainder of this paper is structured as follows: Section 2 discusses the general representation of the model frameworks under RE and BR. The latter includes the description of the forecast heuristics applied while considering the discrete choice mechanism. Section 3 presents the methodology and data used in this study. Section 4 presents a simulation study in which we compare the IRFs obtained from both theoretical models with a VAR model in the case of demand, cost-push, and monetary policy shocks. Finally, Sect. 5 concludes. The technical details and additional results are relegated to “Appendix”.

## Macroeconomic dynamics and expectations

### The hybrid new Keynesian model

The hybrid variant of the baseline NKM with leads and lags is given by the following:

1 $$\begin{aligned} c_t&= \frac{1}{1+\chi } \tilde{E}^{j}_t c_{t+1} +\frac{\chi }{1+\chi } c_{t-1}-\tau (r_t-\tilde{E}^{j}_t \pi _{t+1})+\varepsilon _{c,t} \end{aligned}$$

2 $$\begin{aligned} \pi _t&= \frac{\nu }{1+\alpha \nu } \tilde{E}^{j}_t \pi _{t+1} + \frac{\alpha }{1+\alpha \nu } \pi _{t-1} + \kappa c_t + \varepsilon _{\pi ,t} \end{aligned}$$

3 $$\begin{aligned} r_t&= \phi _{r} r_{t-1} + (1-\phi _{r}) (\phi _{\pi } \pi _t+\phi _c c_t) + \varepsilon _{r,t} \end{aligned}$$

4 $$\begin{aligned} c_t&=y_t \end{aligned}$$

The superscript $j=\{\text{ RE, } \text{ BR }\}$ denotes the RE and BR models, respectively. The corresponding expectations operator is $\tilde{E}^j_t$, which has to be specified for both frameworks in quarterly magnitudes. The variables $c_t$, $\pi _t$, and $r_t$ refer to private consumption (i.e., household expenditure), inflation, and the nominal interest rate, respectively. All the variables are given in gap notation, that is, $s_t={\hat{s}}_t - \bar{s}$ holds, where we consider the deviation of the contemporaneous realization of this variable from its steady-state value denoted by ${\hat{s}}=\{{\hat{c}},{\hat{\pi }},{\hat{i}}\}$ and $\bar{s}=\{\bar{c},{\bar{\pi }},\bar{i}\}$, respectively. In the main text, we omit the expression ‘gap’ to ensure a clear arrangement if it is not necessary otherwise. We assume that the exogenous driving forces follow idiosyncratic shocks $\varepsilon _{s,t}$, which are independent and identically distributed around a mean of zero and a variance of $\sigma ^2_s$ with variables $s=\{c,\pi ,r\}$.

In equation (1), aggregate demand is governed by intertemporal consumption and saving choices. Hence, the representative household smooths consumption over time as a reaction to changes in the real interest rate gap denoted by $r_t-\tilde{E}^j_t \pi _{t+1}$. The consumption behavior is characterized by the inverse of the intertemporal elasticity of substitution ($\tau \ge 0$). The parameter $\chi$ measures the degree of habit formation ($0 \le \chi \le 1$). The idiosyncratic shock $\varepsilon _{c,t}$ can be interpreted as a demand shock.

In equation (2), aggregate supply is affected by inflation dynamics under monopolistic competition and the Calvo-type price-setting scheme. Note here that consumption $c_t$ acts as the driving force of inflation $\pi _t$ in the new Keynesian Phillips curve (NKPC), where its slope is given by the parameter $\kappa \ge 0$. The parameter $\nu$ measures the discount factor ($0< \nu < 1$). The model incorporates the hybrid behavior of the supply curve with the parameter for price indexation ($0 \le \alpha \le 1$). The one-off disturbance $\varepsilon _{\pi ,t}$ stands for a cost-push shock.

The monetary policy is described by the ad hoc Taylor rule in equation (3). The rule includes interest rate smoothing in which the nominal interest rate $r_t$ is a backward-looking variable. The corresponding persistence parameter is $0 \le \phi _{r} \le 1$. The central bank reacts directly to contemporaneous movements in consumption ($\phi _c \ge 0$) and inflation ($\phi _{\pi } \ge 0$). A nominal interest rate shock occurs through $\varepsilon _{r,t}$.

As a market clearing condition, consumption expenditure equals output in the equilibrium, that is, $c_t=y_t$ holds, where the latter denotes the output gap. Hence, equation (4) implies that equation (1) expresses only the standard dynamic IS curve. This becomes even more apparent as equation (4) stands for the national income identity in the absence of private investment, government expenditure, and the trade balance as assumed in our prototype model here. A theoretical-based justification for the appearance of $c_t$ in the NKPC and the Taylor rule given the equilibrium condition is found in the literature; see e.g. Galí (2015) for more details.

### Rational expectations versus bounded rationality

The assumption of rationality suggests that the forward-looking expectations can be described by predictions for consumption and inflation at time $t+1$ in equations (1) and (2), as follows:

5 $$\begin{aligned} \tilde{E}^{RE}_t z_{t+1} = E_t z_{t+1} + E_t\tilde{\varepsilon }_{z,t} \end{aligned}$$

with $z=\{c,\pi \}$ and where $E_t$ denotes the expectation operator conditional on the available information at time *t*. According to the RE hypothesis, agents make rational decisions based on all information available to them. Hence, the expectations are not systematically biased, and forecasting errors are distributed as a normal variable with an expected value of zero, that is, $E_t\tilde{\varepsilon }_{z,t}=0$ holds. The random error term $\tilde{\varepsilon }_{z,t}$ is independent of the future realizations in *z*.

In the BR model, however, agents could make systematic mistakes in their forecasts. Consumption expectations can be modeled employing the following heuristics (cf. Gaunersdorfer et al. 2008 and De Grauwe (2011)):

6 $$\begin{aligned} E^{F}_t c_{t+1}= & {} \bar{c} + \psi _c (c_{t-1}-\bar{c}) \end{aligned}$$

7 $$\begin{aligned} E^{C}_t c_{t+1}= & {} c_{t-1}+\xi _c (c_{t-1}-c_{t-2}) \end{aligned}$$

8 $$\begin{aligned} E^{O}_t c_{t+1}= & {} \frac{1}{2} \cdot [\beta +\delta \lambda _{c,t}] \end{aligned}$$

9 $$\begin{aligned} E^{P}_t c_{t+1}= & {} - \frac{1}{2} \cdot [\beta +\delta \lambda _{c,t}] \end{aligned}$$

Equations (6) to (9) reflect consumers’ forecast heuristics based upon sorting themselves into four groups of forecasters in the absence of the RE hypothesis. We assume that, in general, the steady-state condition $\bar{c}=0$ holds.

The heuristics given by equations (6) and (7) reflect rules-of-thumb because they consist of backward-looking elements. For simplicity, we assume that *fundamentalists* (F) and *chartists* (C) account for professional forecast behavior (i.e., there is an absence of emotional states with limited information). Fundamentalists believe in a convergence of the future value(s) toward the steady-state value $\bar{c}$. The parameter $\psi _c$ controls the speed of convergence ($0 \le \psi _c \le 1$). A quick (slow) movement is observed when $\psi _c$ is close to 0 (1). Chartists form their expectations based on the historical patterns in the time series. Considering the past realization and relation between the first and second lags, this type of agent either extrapolates the previous change in consumption ($\xi _c > 0$) or expects a reversal instead ($\xi _c < 0$). Hence, these heuristics are *technical* in nature. Jang and Sacht (2021) show that consumer confidence is strongly correlated with household expenditure (with a value of 0.66 for the USA and 0.84 for the Euro area, respectively). Consumer confidence is itself also highly persistent over time. These findings suggest that the heuristics in equations (6) and (7) can be viewed as potential candidates to describe the consumers’ expectation formation processes.

For the heuristics given by equations (8) and (9), we follow the specifications proposed by Jang and Sacht (2016, 2021) to quantify the divergence in beliefs. Here, we assume that agents may adopt either an *optimistic* (O) or a *pessimistic* (P) attitude towards movements in future consumption. Hence, both types of agents are uncertain about the future dynamics of consumption and, therefore, predict a subjective mean value of $c_{t+1}$ simply measured by $\beta \ge 0$. However, this type of subjective forecast is generally biased and, therefore, depends on the volatility in consumption (i.e., given by the unconditional standard deviation $\lambda _{c, t} \ge 0$). The corresponding parameter $\delta \ge 0$ measures the degree of divergence in the movement of economic activity. We consider the symmetry of behavioral specifications ($\beta$ and $\delta$) as follows: optimists expect consumption to differ positively from the steady-state value $\bar{c}$ given by the value of $\beta /2$, whereas pessimists expect a negative deviation of the same magnitude. We refer to these heuristics as the *emotional* ones.

Similarly, we incorporate non-RE expectation formation concerning inflation in the BR model where the monetary authority seeks to stabilize inflation via the interest rate channel. An inflation target given by ${\bar{\pi }}$ is announced where the central bank anchors agents’ expectations around this value. Fundamentalists consider this pre-commitment strategy to be fully credible. The corresponding forecasting rule then becomes the following:

10 $$\begin{aligned} E^{F}_t \pi _{t+1}= & {} {\bar{\pi }} \end{aligned}$$

with a target rate for the inflation gap of ${\bar{\pi }}=0$ for simplicity (cf. Jang and Sacht 2016, 2021). Chartists expect the future value of the inflation gap to be given by the expression

11 $$\begin{aligned} E^{C}_t \pi _{t+1}= & {} \pi _{t-1}. \end{aligned}$$

The same heuristics for fundamentalists and chartists as used before (see equations (6) and (7)) are adopted. The differences lie in the assumption that $\psi _{\pi }=\xi _{\pi }=0$ holds. Therefore, we are following the description of the so-called inflation targeters and extrapolators imposed by De Grauwe (2011). Placing these constraints on the heuristics allows us to consider the impact of consumer confidence in isolation.

### Switching mechanism in forecast heuristics

The discrete choice mechanism plays an important role in group behavior under BR. In the following, we describe the mechanism for behavioral choice in the context of the NKM’s core structure mentioned in Sect. 2.1. We consider all the forecast heuristics equations (6) to (11). The market forecast for consumption across the four groups is defined by the following equation:

12 $$\begin{aligned} \tilde{E}^{BR}_{t} c_{t+1} = \sum _{i=1}^{4} (\ \alpha ^{k\{i\}}_{c,t} \cdot E^{k\{i\}}_t c_{t+1}) \end{aligned}$$

with $k=\{ O,P,F,C \}$. The probability $\alpha ^k_{c,t}$ indicates the stochastic behavior of agents who adopt a particular forecasting rule (i.e., out of equations (6)–(9)). More precisely, $\alpha ^k_{c,t}$ can be interpreted as the probability of being an optimist, pessimist, fundamentalist, or chartist for the development of consumption in period *t*.

The selection of the forecasting rules (6) to (9) depends on the forecast performances of each group given by the mean-squared forecasting error $U_{t}^{k}$. The utility for the forecast performances can be simply updated in every period as follows (cf. Brock and Hommes 1997):

13 $$\begin{aligned} U_{t}^{k} = \rho U_{t-1}^{k} \ - \ (1-\rho ) (E_{t-2}^{k}c_{t-1} - c_{t-1})^{2}. \end{aligned}$$

The parameter $\rho$ measures the memory of the four types of agents ($0 \le \rho \le 1$). The polar cases suggest that agents have no memory of past observations ($\rho =0$) or infinite memory ($\rho =1$).

Moreover, economic agents compare the relative accuracy levels of the forecasting methods, and the expectations are revised accordingly. The different types of performance measures can be utilized for $\alpha ^k_{c,t}$ as follows:

14 $$\begin{aligned} \alpha ^{\tilde{k}}_{c,t}&= \frac{\text{ exp }\left( \gamma U_{t}^{\tilde{k}}\right) }{\sum _{i=1}^{4}\text{ exp }\left( \gamma U_{t}^{k\{i\}}\right) } \end{aligned}$$

15 $$\begin{aligned} \alpha ^{C}_{c,t}&= \frac{\text{ exp }\left( \gamma U_{t}^{{C}}\right) }{\sum _{i=1}^{4}\text{ exp }\left( \gamma U_{t}^{k\{i\}}\right) } \cdot \text{ exp }\left[ -\frac{(c_{t-1}-\bar{c})^2}{\varpi }\right] \end{aligned}$$

with $\tilde{k}=\{O,P\}$ and $\varpi >0$. The last term in the heuristic (15) stands for a transversality condition that rules out forever-lasting ‘speculative bubbles’ (cf. Gaunersdorfer et al. 2008 and Hommes 2011).^2^ The parameter $\gamma \ge 0$ denotes the intensity of choice. Equations (12) to (15) have to be adjusted conditional on any expectation formation scenario considered.

The probability of being a fundamentalist is finally given by the following equation:

16 $$\begin{aligned} \alpha ^{F}_{c,t}=1-\sum _{i=1}^{3} \ \alpha ^{\tilde{\tilde{k}}\{i\}}_{c,t} \end{aligned}$$

with $\tilde{\tilde{k}}=\{O,P,C\}$. Again, according to the different scenarios considered, the specification in equation (16) must differ accordingly. Equations (12)–(16) have to be adjusted in the case of the inflation expectation formation process. We assume that the memory parameter $\rho$ remains the same for all heuristics related to consumption and inflation. The inflation-forecasting heuristics also always remain the same; these are given by equations (10) and (11) under BR. For the consumption expectations, we consider specific sets of heuristics. The corresponding choices based on the empirical evidence will be discussed in the following Section.

## Framework for empirical analysis

### Selection of the parameter sets

According to Jang and Sacht (2021), both the BR and RE models are estimated via the simulated method of moment (SMM) approach (cf. Franke et al. 2015 and Jang and Sacht 2016). The BR framework includes different combinations of the forecast heuristics (6) to (9), whereas the rules-of-thumb (10) and (11) hold for inflation.

Notably, both model frameworks differ with respect to their structural representation. For the BR model, the parameters for intrinsic persistence are set to zero. Therefore, in this case $\chi =\alpha =0$ holds. In the RE model, however, we account explicitly for consumption habits and price indexation instead for the following reasons. First, by construction, the hybrid variant of the BR model framework includes more parameters due to the consideration of the forecast heuristics. In this case, we face a tautological argument about the performance of the BR over the RE model. The former, which has more degrees of freedom, could provide a better fit to the data than the latter. To circumvent any potential criticism in this regard, we consider 10 and 10–11 degrees of freedom for the hybrid RE with intrinsic persistence and the BR model with heuristics, respectively. Then, considering similar numbers of parameters would not lead to a bias in our comparison exercise related to the IRFs later on. Second, inertia in the variables is already produced in the BR model based on the heuristics being applied, while a purely forward-looking RE model fails to replicate the persistence in the data. This holds especially in the absence of autocorrelated shocks.^3^ Therefore, the hybrid variant of the RE model should be considered to address the so-called persistence anomaly (Chari et al. 2002).

Indeed, the results of Jang and Sacht (2021) suggest that two specific sets of combinations (which the authors call ‘blocks’) provide the best possible fit of this type of BR model framework to the data compared with other sets of heuristics. For the US economy, the so-called *emotional-fundamental block* (EFB) is the most promising choice. This consists of the forecast heuristics of optimists (8), pessimists (9), and fundamentalists (6) only. For the Euro Area, the highest degree of fitness is observed in the absence of any heuristics linked to an emotional state. Hence, only the expectation formation scheme of fundamentalists (6) and chartists (7) is important. Both heuristics combined represent the so-called *pure-technical block* (PTB).

**Table 1:** Estimation results for the hybrid RE and BR models (excerpt from Jang and Sacht 2021)

Label	US economy		Euro area	
	Hybrid RE	BR EFB	Hybrid RE	BR PTB
$\chi$	1.000	–	1.000	–
	–		–	
$\tau$	0.032	0.371	0.079	0.144
	0.015–0.048	0.222–0.520	0.022–0.136	0.005–0.284
$\sigma _c$	0.554	0.543	0.561	0.413
	0.394–0.714	0.267–0.818	0.430–0.693	0.206–0.619
$\alpha$	0.914	–	0.765	–
	0.803–1.0		0.630–0.900	
$\kappa$	0.030	0.213	0.035	0.152
	0.019–0.040	0.175–0.252	0.021–0.049	0.125–0.178
$\sigma _{\pi }$	0.293	0.240	0.275	0.360
	0.153–0.434	0.018–0.461	0.159–0.390	0.213–0.507
$\phi _{\pi }$	1.573	1.914	1.288	1.593
	1.000–2.228	1.080–2.747	1.0–1.918	1.056–2.129
$\phi _{c}$	0.785	0.709	0.497	0.325
	0.253–1.317	0.011–1.407	0.124–0.870	0.039–0.611
$\phi _{r}$	0.831	0.808	0.604	0.426
	0.766–0.895	0.660–0.956	0.479–0.729	0.229–0.623
$\sigma _{r}$	0.464	0.151	0.421	0.444
	0.133–0.796	0.000–0.417	0.072–0.769	0.078–0.809
$\beta$	–	3.282	–	–
		1.598–4.967		
$\delta$	–	0.531	–	–
		0.000–1.550		
$\psi _{c}$	–	0.951	–	0.762
		0.657–1.244		0.526–0.998
$\xi _{c}$	–	–	–	1.010
				0.574–1.447
*J*	47.33	43.29	56.30	37.96
*p*	0.973	0.989	0.844	0.999

A total of 78 moments (two years) are used based on the SMM approach. The 95% confidence intervals are given with a smaller size. The value of the objective function and the *p*-value are denoted by *J* and *p*, respectively. For the hybrid RE model, the degrees of freedom for the $\chi ^2$ distribution amount to 68, in which the 5% critical value is 88.25. No memory is assumed in the BR scenarios ($\rho =0$). The discount factor $\nu$ is parameterized to 0.99. For the intensity of choice parameter $\gamma =1$ is considered. We set $\varpi$ equal to 1800 (cf. Gaunersdorfer et al. 2008). For a detailed discussion of this kind of parametrization and a description of the SMM approach, see Jang and Sacht (2016, 2021)

Table 1 shows the corresponding estimation results taken directly from Tables 2 and 3 of Jang and Sacht (2021). According to the value of the objective function *J*, which displays the measure of fitness within the SMM approach, the BR model framework exhibits a (slightly) better fit to the data in the Euro area (US economy) than the RE one. In general, lower values of *J* indicate a better fit to the data. Accordingly, the expectations in the US economy are grounded on the consumers’ emotional state, whereas for the Euro area, they are technical in nature. The results for the RE model framework also highlight the importance of backward-looking behavior for the empirical application to both the US economy and the Euro area; the estimates for the habit formation and price indexation parameters in the RE cases are both close (for $\alpha$) or even at their boundary-value of unity (for $\chi$). Jang and Sacht (2021) suggest that the need for a hybrid model variant under RE is disputable because the BR framework exhibits the best description of the data based on their evidence. We refer directly to Jang and Sacht (2016, 2021) for additional details, especially for the description of the SMM approach.

However, the investigation of Jang and Sacht (2021) is not entirely conclusive because, in their paper, there is almost no discussion of macroeconomic dynamics under BR in response to the following different shocks: a demand shock (via the impulse $\varepsilon _{c,t}$ in equation (1)), a cost-push shock (via the impulse $\varepsilon _{{\pi },t}$ in equation (2)), and a monetary policy shock (via the impulse $\varepsilon _{r,t}$ in equation (3)). This is crucial for the study of adjustments in the economy over time for policy analysis. Therefore, this study attempts to compare the IRFs of both frameworks for the US economy and Euro area. To show this, we choose the associated estimated mean values of the parameter sets taken from Table 1 for parametrization. For a robustness check, we compare the simulated IRFs with the empirical ones that stem from a VAR model framework. In the following subsection, we briefly discuss the methodology and data.

### Methodology

We construct the IRFs by observing the economy when presented with an increase in $\varepsilon _{s,\tilde{t}}$ with $s=\{c,\pi ,r \}$ at time $\tilde{t}$ by the value of one. To display the IRFs, we estimate the deviation of the simulated time series from the same time series without shocks. We focus predominately on consumption (cf. equation (1)) and consumer confidence. For the latter, the displayed IRFs are defined as a *measure of dominance* for the heuristics being considered (see below).

The state space representation of the baseline NKM is given by:

17 $$\begin{aligned} \mathbf{A} X_t + \mathbf{B} X^j_{t+1}+ \mathbf{C} X_{t-1} + \mathbf{D} V_t=0, \end{aligned}$$

where $$\mathbf{D}$$ equals the identity matrix; $$\mathbf{X_t} = (c_t,\pi _t, r_t)'$$, $$\mathbf{X^j_{t+1}}=(\tilde{E}^j_t c_{t+1}, \tilde{E}^j_t \pi _{t+1},$$ $$\tilde{E}^j_t r_{t+1})'$$, $$\mathbf{X_{t-1}}=(c_{t-1}, \pi _{t-1},$$ $$r_{t-1})'$$ and $$\mathbf{V_t}=(\varepsilon _{c,t}, \varepsilon _{\pi ,t}, \varepsilon _{r,t})'$$. The latter is a vector of independent and identically distributed random disturbances. These shocks are used to generate the IRFs for the BR, RE and VAR models, respectively.

The reduced-form solution for the RE model reads

18 $$\begin{aligned} \mathbf{X}_t = {\Pi }_0 \mathbf{X}_{t-1} + {\Pi }_1 \mathbf{V}_t \end{aligned}$$

and is obtained by applying the method of undetermined coefficients. The solution matrices ${\Pi }_0$ and ${\Pi }_1$ are computed via the brute force iteration method introduced by Binder and Pesaran (1995).

For the BR model, the corresponding reduced form is given by:

19 $$\begin{aligned} \mathbf{X}_t =-\mathbf{A}^{-1} [\mathbf{B} X^{BR}_{t+1}+\mathbf{C} X_{t-1} + \mathbf{V} _t]. \end{aligned}$$

The forward-looking elements in $X^{BR}_{t+1}$ are replaced by the forecasting heuristics (6)-(11); excluding the expectations on the interest rate, given that $\tilde{E}^{BR}_t r_{t+1}=0$ holds. Since the BR model exhibits a backward-looking structure due to the heuristics (6)-(11), it has to be solved by backward induction.

The VAR in its structural form reads

20 $$\begin{aligned} \mathbf{Q}_0 X_t = \mathbf{Q}_1 X_{t-1} + \ldots + \mathbf{Q}_{\omega } X_{t-\omega } + \mathbf{V}_t \end{aligned}$$

where $\omega$ denotes the maximum number of lags. We assume that this representation of the VAR accounts for the data-generating process based on the underlying relationship of the economic variables, i.e., the output gap, inflation rate and nominal interest rate. The reduced-form solution to (20) is given by

21 $$\begin{aligned} {\mathbf{X}}_t = {\varvec{\tilde{Q}}}_1 X_{t-1} + \ldots + {\varvec{\tilde{Q}}}_{\omega } X_{t-{\omega }} + {\mathbf{Z}}_t \end{aligned}$$

with $\varvec{\tilde{Q}}_h = \mathbf {Q_0^{-1} Q}_h$ for $h=\{1,...,\omega \}$ and $\mathbf{Z}_t = \mathbf{Q}_0^{-1} V_t$. After estimating the VAR model in reduced form via ordinary least squares, we apply the standard Cholesky decomposition according to Sims (1980) to identify the parameters in $Q_0$ and, most importantly, to recover the structural shocks in $V_t$.^4^

Then, the IRF is defined, in general, as follows:

22 $$\begin{aligned} \mathbf{IRF}_{X_t} = E_{t}[{{\mathbf{X}}_{t+1}}|{\nu }_{t}, {\zeta }_{t-1}] - E_{t}[{{\mathbf{X}}_{t+1}}|{\zeta }_{t-1}], \end{aligned}$$

where $\nu _{t}$ is an arbitrary current shock and $\zeta _{t-1}$ refers to historical macroeconomic performance. Note that $\zeta _{t-1}$ is a particular realization of $\Omega _{t-1}$, namely the set containing the information used to forecast $X_{t}$.

In particular, the IRFs under BR show that the response depends on both the persistence of the current shock and the history. Additionally, the simulated trajectory is based on a multivariate nonlinear system. This suggests that the shocks ($\varepsilon _{c, t}, \varepsilon _{\pi , t}, \varepsilon _{r, t}$) have contemporaneous effects on the corresponding variables ($c_{t}, \pi _{t}, r_{t}$), as well as the other macroeconomic variables.

From equation (22), we can understand the different IRFs concerning consumer confidence. For example, according to the EFB scenario for the US economy, we first consider two fractions of groups for two different cases, as follows: i) optimists relative to pessimists *and* fundamentalists versus ii) fundamentalists relative to, let’s say, emotional consumers (optimists *and* pessimists). Both specific fractions are computed in response to the shock. In the second step, we calculate the same configuration in the absence of the shock. In the third and final step, the IRFs are given by the deviation of the relations without the shock from the one where the shock occurs.

For the US economy, we consider two IRFs for consumer confidence in one graph. The IRF labeled “Optimists” indicates the dominance of this group relative to fundamentalists and pessimists if positive realizations above zero are observed. According to the group behavior, fundamentalists and pessimists dominate over optimists in terms of confidence as negative realizations below zero occur. The same type of interpretation holds for the fraction of fundamentalists relative to emotional consumers (i.e., optimists and pessimists). The corresponding IRF is simply labeled “Fundamentalists”. For the Euro area, we consider fundamentalists and chartists only. The trajectory above zero then indicates the dominance of fundamentalists over chartists and vice versa. Concerning the label for the corresponding IRF, we make use of the expression “Dominance of Fundamentalist Strategy” in this case.

Throughout our analysis, we consider the following two subperiods: the *impact* phase (from period $\tilde{t} = 1$ to $t = 10$) and the *convergence* phase (from period $t = 10$ onward). This provides a straightforward interpretation of impulse response analyses because the disturbance by the one-off shock has a strong impact only over a short period. The impact on the dynamics, however, vanishes soon after the shock occurs. We choose period $t=40$ to mark the end of the convergence phase for convenience. In addition, the IRFs from the VAR model framework exhibit only a small to moderate degree of persistence after the impact phase. As we consider the VAR model’s IRF to be a benchmark, we interpret the IRFs from the BR and RE model frameworks in a qualitative way for both phases separately.

To improve the clarity of the figures, we omit the graphical representation of the 95% confidence bandwidth around the mean value in all figures except for the ones linked to the VAR model.^5^ As the confidence bandwidth describes the uncertainty regarding the IRFs, it displays a large impact that was induced by non-autocorrelated shocks for several periods but then gradually converges to the mean IRF as the shocks vanish over time.

The order of the VAR analysis is based on the empirical data. According to the Bayesian information criterion (BIC), we choose a three-quarters lag structure based on quarterly data (see “Appendix B” for more details). Of course, we can include more lags, for example, VAR(8) as a two-year lag, which is consistent with the moment conditions for the estimation of the BR and RE models. However, although including additional lags might improve the fit of the model to the data, it does lead to overfitting, where many parameters are not significant. Hence, we consider three lags to be an appropriate choice for the VAR model and, therefore, make use of the notation ‘VAR(3)’ henceforth.

Although we consider the IRFs stemming from the VAR(3) model as a benchmark, this approach is not protected against valuable counter-arguments. Such statistical models might be criticized simply because they display controversial results. A prominent example is the phenomenon known as the ‘price puzzle’, which is when inflation rises in the case of a contractive monetary policy. We are going to further discuss this issue in our IRF analysis conducted in Sect. 4. Note also that in this study we transform data in gap specification rather than given in levels. Thus, any direct comparison of IRF realizations from a VAR in other studies must be conducted with some caution.

### Data

The data used to compute the IRFs from the VAR(3) model are described as follows. The US data set is retrieved from the Federal Reserve Bank (FED) of St. Louis database (https://fred.stlouisfed.org). The sample spans from 1975:Q1 to 2009:Q4. The output is obtained from the seasonally adjusted real GDP based on billions of chained 2009 dollars. Inflation is measured using the seasonally adjusted consumer price index with 2009 as the base year. The effective federal funds rate is used to measure the short-term nominal interest rate in the USA.

We retrieve the Euro area data set from the 10th update of the Area-Wide Model quarterly database (http://www.eabcn.org/page/area-wide-model; see Fagan et al. (2001)). To remain consistent with the US economy period, the sample covers the period from 1975:Q1 to 2009:Q4. The consumption deflator is used to measure inflation in the Euro area. The short-term nominal interest rate and real GDP are used to measure the gaps in the nominal interest rate and output in the Euro area. The time series in the Area-Wide Model database have the following abbreviations: YER, real GDP; PCD, consumption deflation; and STN, i.e., the short-term nominal interest rate. According to the equilibrium condition $c_t=y_t$, we consider the output gap time series to be a proxy for the private consumption gap (due to limited availability of the consumption data) within our analysis. A standard smoothing parameter of $\lambda =1600$ is used to estimate the trend of the observed data from the Hodrick–Prescott filter for the output, inflation, and nominal interest rate.

Both data sets cover only macroeconomic situations up to 2009:Q4. As is commonly known, the FED started to operate on the zero lower bound from the 16th of December 2008 onwards, when the federal funds rate was set to 0.25%. The European Central bank (ECB) kept the instrument for main refinancing operations stable at a level of 1% until the end of 2009. We omit data points after 2009 to avoid biased results stemming from the Great Recession period. This might be explained by the strong drop in the output gap, while the nominal interest rate hit its zero-lower bound—with only a few observations to be considered. Therefore, the NKPC and the Taylor rule should be reformulated to account for this shift in the monetary policy regime. As examples for such theoretical endeavors, we mention Cochrane (2017) and Galí (2015). In terms of a descriptive comparison of first-order autocorrelations and volatility for different monetary policy regimes in only the USA (including the Great Recession period), we refer directly to Jang and Sacht (2021).

## Simulations and impulse response analysis

**Fig. 1:**
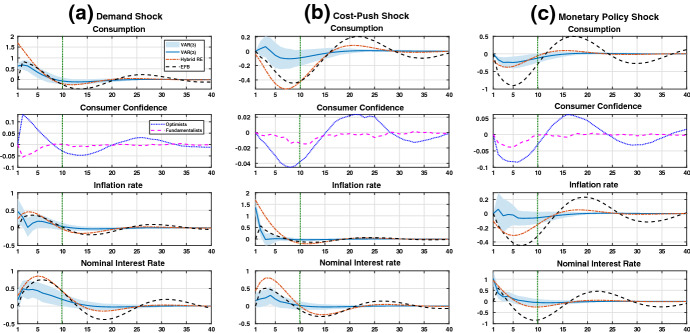
IRFs for the US economy.*Note*: The deviations from the steady state in percentage terms are shown on the vertical axes. The periods, which are presented in quarterly magnitudes, are displayed on the horizontal axes. The IRFs with respect to consumer confidence show the dominance of one group over the other group(s) of consumers. Both theoretical model frameworks are calibrated according to the parameter estimates in the *second* and *third* columns of Table 1. The lag length of 3 in the VAR(3) model is selected based on the Bayesian information criterion (see “Appendix B” for more details). The vertical green dotted line at $t=10$ marks the end (beginning) of the impact (convergence) phase

**Fig. 2:**
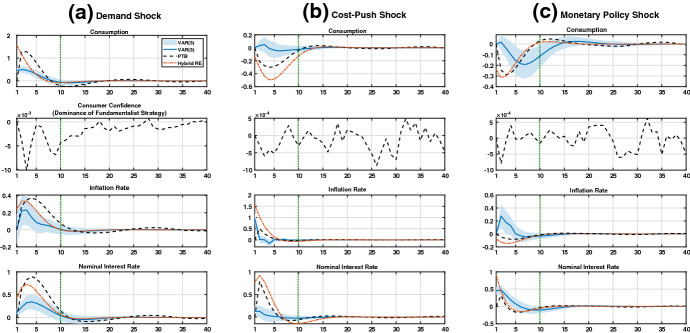
IRFs for the Euro area.*Note* The deviations from the steady state in percentage terms are shown on the vertical axes. The periods, which are in quarterly magnitudes, are displayed on the horizontal axes. The IRFs with respect to consumer confidence show the dominance of one group over the other group(s) of consumers. Both theoretical model frameworks are calibrated according to the parameter estimates in the *fourth* and *fifth* columns of Table 1. The lag length of 3 is selected in the VAR(3) model based on the Bayesian information criterion (see “Appendix B” for more details). The vertical green dotted line at $t=10$ marks the end (beginning) of the impact (convergence) phase

**Fig. 3:**
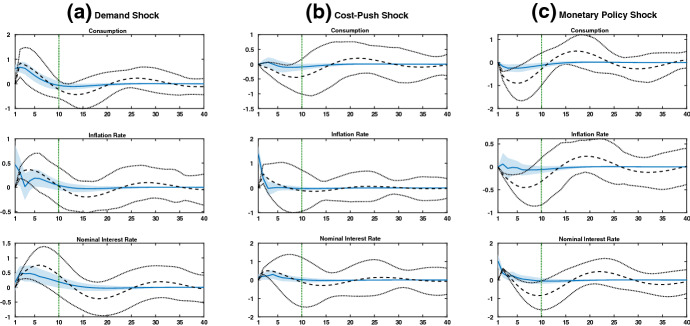
US Economy: IRFs for the BR model (EFB) with Confidence Intervals. *Note* The dashed line shows the IRF for the BR model specification. The dotted lines represent the corresponding upper and lower bounds of the 95% confidence interval. The solid line represents the mean IRF for the VAR(3) model together with its 95% confidence bandwidth, as shown in Fig. 1. The vertical green dotted line at $t=10$ marks the end (beginning) of the impact (convergence) phase

**Fig. 4:**
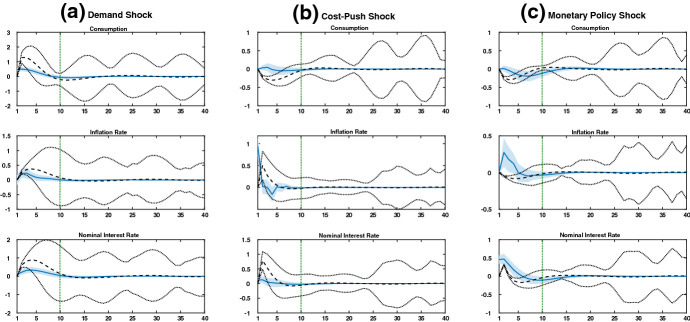
Euro Area: IRFs for the BR model (EFB) with Confidence Intervals. *Note*: The dashed line shows the IRF for the BR model specification. The dotted lines represent the corresponding upper and lower bounds of the 95% confidence interval. The solid line represents the mean IRF for the VAR(3) model together with its 95% confidence bandwidth, as shown in Fig. 2. The vertical green dotted line at $t=10$ marks the end (beginning) of the impact (convergence) phase

In this section, we compare all IRFs under consideration separately for the US economy (Fig. 1) and the Euro area (Fig. 2). Figures 3 and 4 in “Appendix A” show the IRFs for the BR and VAR(3) models with their corresponding 95% confidence bandwidths added. We refer to these specific graphical representations for highlighting the notable observations within our analysis. For completeness, we also display all the model dynamics for the inflation and nominal interest rate in the main body of the text—although these do not appear to be the priority regarding our discussion of the development in consumption linked to confidence.

### US economy

#### Demand shock

The *left* panel (a) of Fig. 1 shows the IRFs of the model when the economy is hit by a positive demand shock. The demand shock is characterized by an exogenous increase in consumption where $\varepsilon _{c,\tilde{t}}>0$ holds. We consider a one-off impulse, where the shock-induced dynamics die out after several periods.

In the impact phase, the demand shock leads to a quantitatively stronger effect on consumption in the RE model compared to the BR model. The IRFs of the latter, therefore, mimic those from the VAR(3). The less pronounced effect following the impact reaction under BR is caused by the dominance of optimists, who expect a subjected mean value of plus $\beta /2=1.62$ (see Table 1). The realized increase in consumption is less than the subjected mean value owing to the existence of pessimists, who consider a negative value of $-1.62$ because of the symmetry in the structure of both forecast heuristics. The fundamentalists are clearly dominated in the impact phase because this group simply expects the (unaltered) previous consumption level to be realized.

In the convergence phase, we observe more fluctuations under BR. Hence, the economy becomes more unstable, which is not in line with the VAR(3) model’s IRF. As the consumption dynamics are closely linked to the change in consumer confidence, we observe the following. While the realization of consumption relies on the dominance of the optimistic group, the volatility in consumption depends on the dominance of the fundamentalists. These observations suggest that over time the dominance of one group over the other alternates: since agents switch from a technical to a more emotionally grounded expectation formation scheme and vice versa, the impact of the shock prevails. This is indeed characterized by a high degree of autocorrelation in consumer confidence, as discussed by Jang and Sacht (2021). The persistence effect is dampened in the RE case without switching. Hence, the BR model is a good approximation for the consumption dynamics in the impact phase, while the opposite is true for the RE model. According to Fig. 1a, this observation is verified as the IRF under BR (RE) lies within the 95% confidence bandwidth generated by the VAR(3) model over the impact (convergence) phase. This resembles the empirical result presented in Jang and Sacht (2021) regarding the fitting of both models to the data, where both values of *J* are, in fact, rather indistinguishable (see the corresponding values for *J* in Table 1).

The IRFs for both theoretical models suggest that they fail to capture the downward movement in the inflation rate at approximately $t=4$. For the nominal interest rate, the IRFs under BR and RE lie outside the VAR(3) model’s 95 % confidence bandwidth following the initial impact effect. At least in the case of the BR model framework, this observation is not necessarily a serious issue. The last entry in Fig. 3a shows that, by direct comparison, the IRFs of the VAR(3) model lie clearly inside the 95% confidence bandwidth stemming from the BR model framework. Note (also in the following) that such an observation cannot be made for the RE model framework because the assumption of the rational expectation hypothesis applies in this case.

#### Cost-push shock

The *middle* panel (b) of Fig. 1 depicts the adjustments in the macroeconomic variables in the case of a non-autocorrelated cost-push shock (i.e., $\varepsilon _{\pi ,\tilde{t}}>0$ holds) in the US and the Euro area. The oil crises in 1973 and 1979 serve as prominent examples of real-life disturbances in this manner. In theory, in the case of a cost-push shock, to target a lower level of inflation after the shock occurs, the central bank must allow for a negative output gap on impact. Therefore, the analysis of such a supply shock is of great interest for formulating (optimal) monetary policy because of the output/inflation trade-off.

For the BR model framework, the forecasting strategy of pessimists dominates in the impact phase. Therefore, the IRFs for consumer confidence labeled "Optimists" and "Fundamentalists" both lie below zero. This suggests that pessimists represent the largest fraction among all groups of boundedly rational agents. Although fundamentalists become more dominated than optimists, the subjective mean value of (minus) 1.62 plays a larger part in anchoring the consumption expectation than the steady-state value does. As a result, the deviation from the VAR (3) model’s IRF is less pronounced than that under the RE model for which future deviations are predicted by construction.

In the convergence phase, we observe a (slightly) better match of the IRFs that stem from the RE model. This finding may be due to the parametrization of the speed of convergence to be considered in fundamentalists’ forecast heuristics. According to our parametrization, $\psi _c=0.95$ holds, indicating an almost purely backward-looking expectation formation scheme applied by this group of agents (see heuristic (6)). Hence, the hump-shaped movement, while primarily caused by the dominance of chartists, is amplified by the forecasting behavior of fundamentalists.

The BR model seems to mimic the real-world consumption dynamics better than the RE model in the impact phase. This holds especially regarding the immediate impact reaction at $\tilde{t}=1$ and the subsequent movement over the first five periods directly afterward. However, the 95% confidence bandwidth shown in the first entry in Fig. 3b indicates that the BR model also accounts for the VAR(3) induced dynamics from period five onwards. Therefore, it can be said that the mean IRF of the VAR(3) model can be reproduced from the BR model framework.

The IRFs under BR show that inflation and interest rate movements come closer to those of the VAR(3) in the impact phase. The opposite is true for those under the RE model (except for the impact reaction in inflation). During and especially at the beginning of the convergence phase, both theoretical models produce slightly more fluctuations than the statistical one does. Again, the observations are in favor of the BR model, i.e., the IRFs of the VAR(3) model lie clearly within the corresponding 95% confidence bandwidths—except for the impact reaction in inflation (see the last two entries in Fig. 3b).

#### Monetary policy shock

An increase in the nominal interest rate by the central bank leads to an increase in the real interest rate because of the Taylor principle. Such a disturbance might represent the behavior of the FED and ECB in the aftermath of the Great Recession. At the time of writing, after a long period of low interest rates close to the zero-lower bound, an increase in the corresponding monetary policy instrument is expected (ECB) or has already been implemented (FED) in response to high inflation rates caused by shocks to the economy due to the COVID-19 pandemic. Moreover, an increase in the interest rate ($\varepsilon _{r,\tilde{t}}>0$) acts as a negative monetary policy shock because both output and inflation decrease upon impact. This is observed by inspecting the corresponding IRFs induced by both theoretical models.

The *right* panel (c) of Fig. 1 shows the adjustments in consumption and consumer confidence over time for the US economy. The IRFs based on the VAR(3) model deviate (significantly) from the other two in the impact and convergence phases. In this scenario, the movement under RE comes close to the one predicted by the real data, especially in the impact phase (excluding the reaction in $\tilde{t}=1$). In the scenario under BR, like in the case of a cost-push shock, the group of pessimists clearly dominates, as according to the second entry in Fig. 1c, the corresponding IRFs for consumer confidence are below the zero line. This is not surprising because the forecast performance of pessimists (cf. equation (13)) seems to attain the highest value as a result of the negative shock. Hence, a further negative deviation from the steady state is expected and members of the other two groups become pessimists. This translates into a strong negative reaction in terms of consumption (as shown in the first entry in Fig. 1c) after the shock occurs.

The previous explanation is tied into the behaviors of optimists. The corresponding IRF, which displays the dominance of this group, alternates heavily over time. We observe a large trough and peak in the impact phase and at the beginning of the convergence phase, respectively. The upswing in consumer confidence after period 5 is grounded on the fact that the negative monetary policy impulse gradually vanishes. Consequently, the forecast performance of the optimists improves. The strong increase in confidence induced by a large amount of switching to the optimistic group leads to a boom period as consumption recovers after periods of high interest rates being observed in the impact phase.

The high degree of fluctuations in the IRF under BR is linked to the development in the level of fundamentalists’ dominance, who apply an almost purely backward-looking expectation formation scheme (note, again, that $\psi _c=0.95$ holds). As consumption equals its steady-state value around period $t = 11$ under BR, no group of bonded rational agents dominates the other, as shown by the intersection of the IRFs that resemble the dominance of optimists and fundamentalists at that exact point in time. Overall, the monetary policy in the US economy creates high fluctuations over the business cycle in the absence of RE. This, however, is at odds with the observed development based on the real data. This holds, as the impact reaction of the VAR(3) model is not covered by possible realizations induced by the BR model, except for the realization on impact. This is judged by the displayed confidence bandwidth in the first entry in Fig. 3c. However, as in the case of the cost-push shock, from period five onwards, there is the possibility for the BR model to mimic the IRFs induced by the VAR (3) model.

Indeed, the noticeable movement in consumption based on the VAR(3) model might be linked to the observed upward swing in the inflation rate. As aforementioned, an increase in inflation as a response to a negative monetary policy shock is known as the ‘price puzzle’. Observations in this regard have been made by Christiano et al. (2005), among others. The puzzle might hold due to indeterminacy (Lubik and Schorfheide 2003) and the fact that VAR models lack a minimal set of structural assumptions (Estrella 2015). Not surprisingly, the IRFs under BR and RE move in the opposite direction in the impact phase, whereas the ones under BR come very close but exhibit, once again, a higher fluctuation degree in the convergence phase. Based on the second entry in Fig. 3c, the mean VAR(3) model’s IRFs lie inside the confidence bandwidth induced by the BR model framework only to the end of the impact phase going forward. However, linked to our previous statements, these observations might question the VAR(3) model framework rather than the BR framework in the case of a monetary shock due to the ‘price puzzle’.

Regarding the interest rate dynamics, the result does not seem to develop well for the BR model. While the IRFs from the RE model nearly perfectly overlap with the ones from the VAR(3) model, the BR model undershoots the corresponding impact reaction and exhibits a large degree of volatility in comparison. In addition, the VAR(3) model’s mean IRFs start to fit into the confidence bandwidth of the BR model only at the beginning of the convergence phase (see the last entry in Fig. 1c).

### Euro area

#### Demand shock

The *left* panel (a) of Fig. 2 shows the outcome for the Euro area. In the impact phase, we observe fluctuations in the dominance of chartists over fundamentalists. This is indicated by the IRF for consumer confidence, which is below zero (see the second entry in Fig. 2a) This leads to an increase in consumption over time, as in the RE case. The IRFs for both the BR and RE models differ significantly from those for the VAR(3) model. This can be explained by the high weight on the backward-looking expectation formation in both theoretical frameworks (i.e., $\chi =1$, $\psi _c=0.76$ and $\xi _c=1.01$ given in Table 1). According to Fig. 4a, the IRFs for the VAR(3) model lie within the BR model’s 95% confidence bandwidth from period three onwards.

Consumption dynamics become more volatile under BR, which holds because of the dominance of chartists in the impact phase. Indeed, this group extrapolates into the future under the consideration of the past realization of consumption up to the second lag. This dominance translates into the highest peak (in period $t = 3$) and lowest trough (in period $t = 11$) in consumption, which coincides with the fact that fundamentalists are greatly dominated around these periods. Therefore, in these time periods, the IRF (to be shown in the second entry in Fig. 2a) is substantially below zero. The increase in the relative fraction of fundamentalists from the beginning of the convergence phase contributes to a decrease in volatility over time. However, in the absence of RE, volatility in consumption prevails in the BR model framework, while the opposite holds for the RE model.

Regarding the inflation and interest rate dynamics, the IRFs of the BR model deviate strongly from those obtained by the VAR(3) model—at least for the later stages of the impact phase. For the first periods, we instead observe a (pronounced) side-by-side movement in the two models’ IRFs as both originate very close to each other. This holds especially in the case of inflation rate dynamics, for which we discover an overlapping development of this variable over the first three periods. The last two entries in Fig. 4a show that the IRFs of the VAR(3) model fit well into the confidence bandwidths under BR for both cases. An exception is given by the initial impact reaction in the case of nominal interest rate dynamics.

#### Cost-push shock

According to the *middle* panel (b) of Fig. 2, like in the US case, for the Euro area we observe that both theoretical models fail to replicate the upward movement in the VAR(3) model’s IRFs after impact. However, in the impact and convergence phases, the IRFs under BR come close to those in the VAR(3) model. This observation can be explained by the ongoing fluctuation in consumer confidence. As fundamentalists and chartists dominate each other alternately over time, this leads to a small degree of fluctuation in consumption around the steady state in the convergence phase, as shown by the rough oscillation of the IRF around zero in the second entry of Fig. 2b.

The trough in the dynamics of consumption at the beginning of the impact phase under BR match the periods of dominance by chartists (e.g., around $t = 3$ to $t = 5$). Note here that the exploration parameter $\xi _c$ is estimated to be in unity. This indicates a purely backward-looking expectation formation scheme according to chartists’ forecast heuristic (7). By comparison, the speed of convergence in the fundamentalists’ rule-of-thumb (6) is high but in unequal unity with $\psi _c=0.76$ for the Euro area. For the RE model framework, the discrepancy in the IRF is large in the impact phase, while the dynamics quickly fade out in the convergence phase, as no switching mechanism is assumed.

For the inflation rate, we observe that the IRFs under BR mimic those from the VAR(3) model well, except for the impact reaction. The RE model framework exhibits a stronger reaction over the impact phase, as expected by the statistical model. In the convergence phase, all three IRFs are almost indistinguishable. Regarding the interest rate, the IRFs under BR and RE exhibit a much stronger reaction over the impact phase than what can be expected based on the real data. Upon inspection of the last two entries in Fig. 4b, it becomes apparent that the mean IRFs of the VAR(3) model lie within the BR model’s 95% confidence bandwidth from period three forward.

#### Monetary policy shock

The IRF of the VAR model displayed in the *right* panel (c) of Fig. 2 shows an increase in consumption in the impact phase for the Euro area scenario. However, as theoretically expected by the core structure of the NKM, household expenditure drops under BR and RE, we observe a peak at approximately period $t = 2$ given the real data. As a result, the IRFs that stem from both theoretical models are the direct opposite of those of the VAR(3) model. Further, the VAR(3) model’s IRFs also exhibit a high degree of peaks and troughs, which leads to a longer convergence time back to the steady state. The higher rate of convergence under BR and RE can be explained by the purely backward-looking expectation schemes applied in both models. The dominance of chartists at the beginning of the impact phase, therefore, translates into the trough at approximately period $t = 2$ for consumption under BR. This is followed by an upswing in consumption starting at period $t=4$, as fundamentalists now dominate chartists, as observed in the second entry of Fig. 2c. As consumer confidence fluctuates around zero from the beginning of the convergence phase onwards, the corresponding fluctuations in consumption are mitigated under BR.

The analysis of the IRFs for the Euro area reveals that both model frameworks fail to capture real economic developments, including inflation and nominal interest rate dynamics (with the expectations of an overlap in the impact reactions for the BR and VAR(3) cases regarding consumption and inflation). This is confirmed by checking on all entries in Fig. 4c, where the mean IRFs of the VAR(3) model do not fit into the BR model’s 95% confidence bandwidth until approximately period $t=5$. As in the US economy, this raises the question of potential misspecification in the model structure of the VAR(3) model. We also find the occurrence of the ‘price puzzle’ in inflation dynamics. It is worthwhile, however, to check whether the consideration of more elaborate specifications of both theoretical model frameworks, in terms of additional structural equations, might solve the mismatch of the IRFs. The same may be especially true for the BR model introducing forecast heuristics different from those discussed in this study, which should also be considered.

## Conclusion

The discussion of the most suitable type of economic model for describing the adjustment over the business cycle has become more crucial since the period of worldwide distress after 2008. Perhaps we could depart from perfect rationality to find a good approximation to reality. Given this view, an alternative approach to macroeconomic dynamics has gained importance, and all the specific types of forecast heuristics under bounded rationality have led to a variety of stylized models being used for policy analysis in the absence of RE.

In this study, we calibrate two versions of the baseline NKM based on the results of the parameter estimates provided by Jang and Sacht (2021). We then analyze the IRFs for the RE and BR models with a focus on the US economy and the Euro area. In the case of the BR model, we assume that specific forecasting heuristics are applied in the expectation formation. Our analysis highlights the importance of relevant policies during the transition period because a policymaker faces different dynamic consumption patterns based on the degree of rationality. To check the plausibility of our results, we compare the different IRFs to the ones obtained from a standard VAR model based on real data.

Considering the dynamics in all variables, it can be stated that a BR model with a heuristic-induced switching process can qualitatively provide a good fit to the data that is equivalent to a hybrid RE model. The focus of our study is on the development of consumption over time. Our observations reveal that, in the majority of the cases, the BR model is supported by the empirical data. This holds in terms of the smaller deviations of the corresponding IRFs to those from the VAR model compared with the framework under RE. Additionally, it is worth mentioning that the mean IRFs from the VAR lie within the 95% confidence bandwidth induced by those of the BR. However, this holds only to a certain extent, as both theoretical models mostly fail to reproduce the impact reactions induced by the VAR model, with the BR model having a (slightly) better outcome. The fluctuations in consumer confidence, as a measure of the dominance of one group of boundedly rational agents over the others, mainly explain the degree of persistence in consumption. This is also confirmed by the robustness exercise of Jang and Sacht (2021), which considers different US monetary policy regimes. Without a switching mechanism in expectation formation, the hybrid version of the model under RE with lead and lags fails to describe the hump-shaped consumption behavior.

Although the previous statement remains valid for a positive demand and (partly for) a cost-push shock, both models generate IRFs that are at odds with the prediction of the model regarding consumption in the case of a negative monetary policy shock—in both the US and Euro area scenarios. This raises the question of whether this holds due to a severe model misspecification of the VAR(3) and/or both theoretical frameworks. In the former case, choosing a different strategy for the identification of the structural shocks in the VAR model might be a valuable option—in comparison with a rather atheoretical one like the Choleski decomposition being applied in this study. For the latter, this issue can be overcome by allowing a more elaborate model structure combined with different forecast heuristics (other than the ones considered here) in the BR model. Our results indicate that further research on the impact of the different kinds of shocks under the incorporation of rule-of-thumb behavior into macroeconomic dynamics is needed. We leave this to future research and claim that our analysis of IRFs stands out as a point of reference in this regard.

## Appendix B: selection of the lag length in the VAR model

For selecting the lag length in the VAR model, we apply both the Akaike and Bayesian information criterion (for which the corresponding abbreviations AIC and BIC hold), respectively. The formulations of these criteria read as follows (see Ivanov and Kilian (2005)):

23 $$\begin{aligned} IC^s(\omega )=\ln \left| {\overline{\sum }}(\omega )\right| + \Theta ^s (K^2\omega ) \end{aligned}$$

with $s=\{A,B\}$. The AIC [BIC] denoted by $IC^A$ [$IC^B$] is defined for $\Theta ^A=2/N$ [$\Theta ^B=\ln (N)/N$]. *K* and *N* stand for the dimension and the number of observations, respectively. ${\overline{\sum }}$ is the quasi-maximum likelihood estimate of the innovation covariance matrix $\sum$. The lag order estimate ${\hat{\omega }}$ is chosen to minimize the value of the criterion function for $\{\omega : 1 \le \omega \le {\overline{\omega }}\}$, where ${\overline{\omega }}\ge \omega _0$ holds.

We are interested in the determination of the appropriate lag length in the VAR model for which the unwanted outcome of over- and/or underfitting is considered to be unlikely. Therefore, we compute the values for $IC^A$ and $IC^B$ based on the VAR model with a lag length ranging from one to ten. Again, according to definition (23), the model specification with the lowest values is preferred.

**Table 2:** Numerical evaluation of the values based on the AIC and BIC criteria

Model	US Economy		Euro Area	
	$IC^A$	$IC^B$	$IC^A$	$IC^B$
VAR(1)	1210.5	1245.7	803.4	838.7
VAR(2)	1196.5	1258.2	778.4	840.2
**VAR(3)**	1173.2	**1235.0**	**764.1**	**825.9**
VAR(4)	**1159.4**	1247.7	773.5	861.8
VAR(5)	1160.4	1301.6	775.8	917.0
VAR(6)	1132.3	1300.0	730.6	898.2
VAR(7)	1124.9	1319.1	732.5	926.7
VAR(8)	1124.4	1345.1	732.1	952.7
VAR(9)	1113.8	1360.9	728.1	975.2
VAR(10)	1097.9	1371.5	716.5	990.1

VAR($\omega$) denotes the VAR model under consideration of the lag length with $\omega =\{1\ldots 10\}$. The values based on the AIC and BIC are given by $IC^A$ and $IC^B$, respectively, and computed via definition (23). The numbers in bold type denote the corresponding lowest values

Table 2 shows the numerical outcome regarding $IC^A$ and $IC^B$. Based on these results, we find ${\hat{\omega }}=3$ and judge the VAR(3) model to be the statistical model specification used for our comparison exercise throughout the paper. Note that the AIC suggests that four lags are desirable for the improvement of the model fit in the US economy case. This might be explained by the penalty term for the number of parameters in the model is larger in BIC than in AIC. However, the higher number of lags may also increase the risk of overfitting. Hence, we choose the VAR(3) model to be our benchmark model.

## Footnotes

1 Notably, the discrete choice approach has been widely used in financial economics over the past two decades. Seminal contributions have been made by Brock and Hommes (1997) and Gaunersdorfer et al. (2008), among others. Moreover, the study of De Grauwe (2011) might be acknowledged as one of the most influential early theoretical contributions to dynamic stochastic general equilibrium (DSGE) models under bounded rationality. See also Jang (2020) who applies the discrete choice approach to an open-economy DSGE model.

2 This transversality condition is crucial because, according to heuristic (7), chartists react to the historical pattern of consumption for up to two lags but not to the steady state. It follows that as this group exhibits the best performance among all groups, the corresponding forecasting strategy becomes the dominant one. In this case, we observe an ongoing deviation of consumption from its steady-state value. We are interested in the analysis of shocks under stability; hence, we account for this type of penalty term in equation (15) with the value of $\varpi =1800$ as imposed by Gaunersdorfer et al. (2008).

3 In fact, we consider non-autocorrelated shocks only to reduce the number of parameters to be considered for parametrization. This accounts for a higher accuracy of the estimates in terms of parameter identification. Persistence in the IRFs induced by both theoretical models is nevertheless guaranteed via the inclusion of heuristics (BR) or an existing lag structure (RE).

4 Procedures for recovering the structural shocks in the VAR model are well known. We refer to the corresponding literature, e.g., Chapter 2 (especially Sect. 2.3.2) in Lütkepohl (2005).

5 For an eye-ball comparison exercise, we display the mean IRFs and 95% confidence bandwidths for the VAR and BR models in Figs. 3 and 4, which can be found in “Appendix A”. See Sect. 4 for more information. We decompose the graphical outcome in the main body of the text and in “Appendix” to improve the clarity and visibility.
